# Aberrant Expression of Histone Deacetylases 4 in Cognitive Disorders: Molecular Mechanisms and a Potential Target

**DOI:** 10.3389/fnmol.2016.00114

**Published:** 2016-11-01

**Authors:** Yili Wu, Fei Hou, Xin Wang, Qingsheng Kong, Xiaolin Han, Bo Bai

**Affiliations:** ^1^Department of Psychiatry, Jining Medical UniversityJining, China; ^2^Collaborative Innovation Center for Birth Defect Research and Transformation of Shandong Province, Jining Medical UniversityJining, China; ^3^College of Science, Qufu Normal UniversityJining, China; ^4^Department of Biochemistry, Jining Medical UniversityJining, China

**Keywords:** HDAC4, cognitive function, cognitive impairment, neurodegenerative diseases, mental disorders

## Abstract

Histone acetylation is a major mechanism of chromatin remodeling, contributing to epigenetic regulation of gene transcription. Histone deacetylases (HDACs) are involved in both physiological and pathological conditions by regulating the status of histone acetylation. Although histone deacetylase 4 (HDAC4), a member of the HDAC family, may lack HDAC activity, it is actively involved in regulating the transcription of genes involved in synaptic plasticity, neuronal survival, and neurodevelopment by interacting with transcription factors, signal transduction molecules and HDAC3, another member of the HDAC family. HDAC4 is highly expressed in brain and its homeostasis is crucial for the maintenance of cognitive function. Accumulated evidence shows that HDAC4 expression is dysregulated in several brain disorders, including neurodegenerative diseases and mental disorders. Moreover, cognitive impairment is a characteristic feature of these diseases. It indicates that aberrant HDAC4 expression plays a pivotal role in cognitive impairment of these disorders. This review aims to describe the current understanding of HDAC4’s role in the maintenance of cognitive function and its dysregulation in neurodegenerative diseases and mental disorders, discuss underlying molecular mechanisms, and provide an outlook into targeting HDAC4 as a potential therapeutic approach to rescue cognitive impairment in these diseases.

## Introduction

Histone deacetylases (HDACs), accompanying with histone acetyltransferases (HATs), are implicated in chromatin remodeling and subsequent transcription regulation by controlling the status of histone acetylation. Histone acetylation makes chromatin conformation more relaxed, facilitating gene transcription, whereas histone deacetylation induces a condensed chromatin conformation repressing gene transcription. By controlling the status of histone acetylation, HDACs are involved in diverse physiological and pathological processes. Moreover, the function of HDACs is not limited to the histone deacetylation. Recent evidences suggest that HDACs may also contribute to the deacetylation of non-histone proteins (Lardenoije et al., 2015). In addition, HDACs also have deacetylase-independent functions, such as histone deacetylase 4 (HDAC4) (Lardenoije et al., 2015; Han et al., 2016).

HDAC4 is highly expressed in brain (Grozinger et al., 1999; Bolger and Yao, 2005; Darcy et al., 2010). It plays a key role in the maintenance of cognitive function and its alteration is associated with cognitive impairment in both age-related neurodegenerative diseases (e.g., Alzheimer’s disease, AD) and development-related mental disorders (e.g., autism). Therefore, the role of HDAC4 in cognitive function, its dysregulation in cognitive impairment-related neurodegenerative diseases and mental disorders, and underlying mechanisms are discussed in this review.

## HDAC4 and HDACs

### HDACs Classification

Eighteen human HDACs are identified and classed into four groups based on their homology to yeast HDACs (Didonna and Opal, 2015). Class I HDACs, consisting of HDAC1, 2, 3, and 8, are homologous to yeast RPD3 while class II HDACs have high identity to yeast HDA1, consisting of HDAC4, 5, 6, 7, 9, and 10. According to the protein structure and motif organization, class II HDACs are further divided into two subclasses, class IIa with HDAC4, 5, 7, and 9, and class IIb with HDAC6 and 10. Class III HDACs, named sirtuins, including SIRT1-7, are homologous to yeast SIR2. Compared with zinc-dependent HDACs of class I and class II, class III HDACs are nicotinamide-adenine-dinucleotide (NAD)-dependent. HDAC11 is the only member of Class IV, which is also a Zn-dependent HDAC.

### The *HDAC4* Gene and Protein

The human *HDAC4* gene, located on chromosome 2q37.3, spans approximately 353,480 bp encoding HDAC4 protein with 1084 amino acids. HDAC4 shuttles between cytoplasm and nucleus depending on signal transduction-related phosphorylation status of HDAC4 (Mielcarek et al., 2013). Normally, phosphorylated HDAC4 retains in the cytoplasm, while dephosphorylated HDAC4 is imported into the nucleus (Nishino et al., 2008).

**FIGURE 1:**
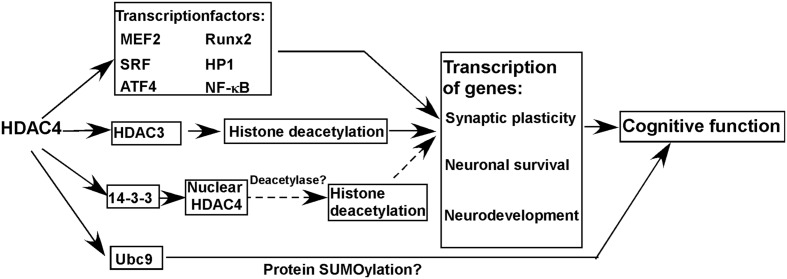
**Histone deacetylase 4 (HDAC4) in cognitive function and molecular mechanisms.** HDAC4 is a global regulator of the transcription of genes involved in synaptic plasticity, neuronal survival, and neurodevelopment by interacting with multiple proteins, which is essential for the maintenance of normal cognitive function. Moreover, HDAC4 may function to regulate protein SUMOylation via interacting with Ubc9 contributing to the maintenance of cognitive function. Solid line and dash line represent confirmed and possible mechanisms, respectively.

Histone deacetylase 4 protein consists of a long N-terminal domain and a highly conserved C-terminal catalytic domain. The deacetylase activity of HDAC4 is almost undetectable although it has a conserved C-terminal catalytic domain, which might be caused by a substitution of tyrosine to histidine in the enzyme active site (Lahm et al., 2007). However, HDAC4 does play an important role in the regulation of gene transcription via different ways (**Figure 1**). First, HDAC4 interacts with multiple transcriptional factors [e.g., myocyte enhancer 2 (MEF2), runt related transcription factor 2 (Runx2), serum response factor (SRF), heterochromatin protein 1(HP1), nuclear factor kappa B (NF-κB)] regulating gene transcription (Sando et al., 2012; Ronan et al., 2013). Although HDAC4 per se lacks deacetylase activity, it may be involved in histone deacetylation-mediated transcriptional regulation via interacting with HDAC3, another member of the HDAC family with deacetylase activity (Grozinger et al., 1999; Lee et al., 2015). For example, Lee et al. (2015) showed that HDAC4 is crucial for HDAC3-mediated deacetylation of mineralocorticoid receptor, which could be inhibited by class I HDAC inhibitor but not class II HDAC inhibitor, indicating that HDAC4 is implicated in protein deacetylation via the deacetylase activity of HDAC3. Moreover, the deacetylase activity of HDAC4 needs to be further investigated by multiple approaches as it is not convincing by the *in vitro* assay from one study (Lahm et al., 2007). As the nuclear localization of HDAC4 is regulated by its interaction with14-3-3, it is possible that the alteration of nuclear HDAC4 mediated by tyrosine 3-monooxygenase/tryptophan 5-monooxygenase activation protein (14-3-3) is involved in transcriptional regulation by its deacetylase activity (Nishino et al., 2008). A recent study suggests that HDAC4 may function to regulate protein SUMOylation via interacting with SUMO-conjugating enzyme Ubc9 (Ubc9), a SUMO E2-conjugating enzyme, contributing to memory formation (**Figure 1**) (Schwartz et al., 2016).

## HDAC4 in Cognitive Function and Molecular Mechanisms

A growing body of evidence indicates that the homeostasis of HDAC4 is crucial for the maintenance of cognitive function by regulating genes involved in synaptic plasticity, neuronal survival and neurodevelopment (**Figure 1**) (Schwartz et al., 2016).

### HDAC4 and Synaptic Plasticity

Histone deacetylase 4 interacts with multiple transcription factors (e.g., MEF2, Runx2, SRF, HP1), 14-3-3, HDAC3 etc. regulating the transcription of genes involved in synaptogenesis, synaptic plasticity and neurodevelopment, such as activity regulated cytoskeleton associated protein (*Arc*) and protocadherin (*Pcdh10*) (**Figure 1**) (Grozinger et al., 1999; Sando et al., 2012; Ronan et al., 2013; Rashid et al., 2014; Lee et al., 2015; Sharma et al., 2015; Krishna et al., 2016; Nott et al., 2016). First, nuclear HDAC4 represses the expression of constituents of synapses leading to the impairment of synaptic architecture and strength in mice (Sando et al., 2012). In addition, mice carrying a gain-of-function nuclear HDAC4 mutant exhibit deficits in neurotransmission, learning and memory (Sando et al., 2012). On the other hand, silencing HDAC4 expression does result in the impairment of synaptic plasticity, and learning and memory deficits in both mice and Drosophila (Kim et al., 2012; Fitzsimons et al., 2013). A proteomics analysis indicates that HDAC4 is a regulator of proteins involved in neuronal excitability and synaptic plasticity, which are differentially expressed in normal aging subjects and AD patients and associated with memory status (Neuner et al., 2016). A recent study showed that HDAC4 interacts with Ubc9 during memory formation, while the reduction of Ubc9 in adult brain of Drosophila impairs long-term memory, suggesting that the role of HDAC4 in memory formation may be associated with the regulation of protein SUMOylation (**Figure 1**) (Schwartz et al., 2016).

Above evidence suggests that HDAC4 homeostasis is crucial for the maintenance of synaptic plasticity and cognitive function, i.e., both HDAC4 elevation and reduction lead to cognitive deficits. It is not surprised that both up-regulation and down-regulation of HDAC4 impairs synaptic plasticity and memory function as previous studies have demonstrated that a number of molecules play a dual role in synaptic plasticity and memory function. For example, both overexpression and disruption of regulator of calcineurin 1 (RCAN1) leads to synaptic impairment and memory deficits in Drosophila and mice (Chang et al., 2003; Hoeffer et al., 2007; Chang and Min, 2009; Martin et al., 2012). Moreover, the bidirectional alterations of HDAC4 may differentially disrupt the balance between HDAC4 and its interacting partners leading to synaptic impairment and memory deficits as HDAC4 is implicated in multiple signaling pathways by interacting with many functional proteins (**Figure 1**). However, the underlying mechanisms need to be further investigated.

### HDAC4 and Apoptosis

Neuronal apoptosis is a major mechanism linking to cognitive deficits. In addition to synaptic plasticity, HDAC4 is also involved in neuronal apoptosis. For example, HDAC4 interacts with NF-κB repressing proapoptotic gene expression, and it also inhibit ER stress-induced apoptosis by interacting with activating transcription factor 4 (ATF4), a key transcriptional factor in ER stress response (**Figure 1**) (Zhang et al., 2014; Vallabhapurapu et al., 2015). Moreover, Majdzadeh et al. (2008) showed that HDAC4 overexpression protects mouse cerebellar granule neurons (CGNs) from apoptosis by inhibiting cyclin dependent kinase 1 (CDK1) activity. Consistently, upregulation of HDAC4 by a NMDAR antagonist protects mouse hippocampal neurons from naturally occurring neuronal death, whereas HDAC4 reduction promotes neuronal apoptosis during development (Chen and Cepko, 2009). Sando et al. (2012) further demonstrated that HDAC4-C-terminal is crucial for rescuing HDAC4 knockdown-induced cell death and reduction of synaptic strength in mouse brains. However, Bolger and Yao (2005) showed that increased expression of nuclear-localized HDAC4 promotes neuronal apoptosis in mouse CGNs, while down-regulation of HDAC4 protects neurons from stress-induced apoptosis. The conflicting results may be caused by different cell types and culture conditions. Majdzadeh et al. (2008) cultured CGNs for 4-5 days before transfection, while Bolger and Yao (2005) transfected CGNs immediately after cell isolation. Although CGNs were used in both studies, the maturation status of neurons when they were transfected may have significant effects on the conflict results. Moreover, short-term and long-term protein overexpression may have opposite effects. For example, a previous study showed that RCAN1 plays an opposite role in neuronal apoptosis at different culture stages, which may be associated with aging or maturation processes (Wu and Song, 2013).

### HDAC4 and Brain Development

Histone deacetylase 4interacts with multiple transcriptional factors, repressing the transcription of genes involved in neurodevelopment (**Figure 1**) (Sando et al., 2012; Ronan et al., 2013). Moreover, HDAC4 may be implicated in neurodevelopment via interacting with HDAC3 which is necessary for brain development (Norwood et al., 2014). In human, both HDAC4 deletion and duplication lead to mental retardation and intelligence disability, suggesting that HDAC4 plays an important role in neurodevelopment which directly links to cognitive function (Shim et al., 2014).

## Aberrant HDAC4 Expression/Localization in Neurodegenerative Diseases

Cognitive decline, in particular, learning and memory deficits, is a characteristic of neurodegenerative diseases [e.g., Alzheimer’s disease (AD), Huntington’s disease (HD), and Parkinson’s disease (PD)] which are associated with synaptic dysfunction and synaptic and neuronal loss. A large body of evidence indicates that aberrant HDAC4 expression and subcellular distribution may contribute to the cognitive decline in patients with neurodegenerative diseases (**Table 1**). First, increased HDAC4 expression was observed in prefrontal cortex of aged individuals, while aging is the major risk factor of neurodegenerative disorders (Sharma et al., 2008). In addition, a more recent study showed that HDAC4 is a global regulator of memory deficits with age (Neuner et al., 2016). Moreover, HDAC4 is involved in the regulation of SIRT1 which is implicated in both aging and memory process in rats (Sasaki et al., 2006; Sommer et al., 2006; Quintas et al., 2012; Han et al., 2016). Furthermore, as mentioned in the section 2.1, HDAC4 homeostasis is crucial for the maintenance of cognitive function, i.e., both HDAC4 elevation and reduction lead to cognitive deficits.

**Table 1:** The alteration of histone deacetylase 4 (HDAC4) in cognitive-related disorders.

Disease	Subject	tHDAC4	nHDAC4	cHDAC4	Beneficial effects	DNA	Reference
AD	Human		Up				Shen et al., 2016
AD	Mouse	Up					Anderson et al., 2015
AD	Mouse		Up				Sen et al., 2015
FTLD	Human			Up			Whitehouse et al., 2015
HD	Mouse	No			Down		Mielcarek et al., 2013
HD	Mouse	Down					Quinti et al., 2010
ATM	Mouse	Up					Li et al., 2012
ASD	Human	Up^∗^					Nardone et al., 2014
BDMR	Human					Mutation	Williams et al., 2010
Depression	Human	Up^∗^					Hobara et al., 2010
Depression	Mouse	Up^∗^					Sarkar et al., 2014
Depression	Mouse	Up^∗^					Sailaja et al., 2012
Schizophrenia	Human	No^∗^					Sharma et al., 2008
Schizophrenia	Human					SNP	Kim et al., 2010


*tHDAC4, total HDAC4; nHDAC4, nuclear HDAC4; cHDAC4, cytoplasmic HDAC4; no, no change; ^∗^, mRNA.*

### HDAC4 in Alzheimer’s Disease

Alzheimer’s disease is the most common form of neurodegenerative disorders in the elderly leading to dementia. Progressive memory loss is the clinical characteristics of AD. Neuritic plaques, neurofibrillary tangles and neuronal loss are the neuropathological hallmarks of AD. Amyloid β (Aβ) and phosphorylated microtubule associated protein tau (Tau) are the major components of neuritic plaques and neurofibrillary tangles, respectively, while apoptosis is a major mechanism of neuronal loss (Wu et al., 2014).

The nuclear expression of HDAC4 is markedly increased in brains of AD patients, while the alteration of total HDAC4, including both cytoplasmic and nuclear HDAC4, is not conclusive (Shen et al., 2016). However, the expression of HDAC4 was significantly increased in AD model mice (Anderson et al., 2015). Moreover, ApoE4, the only confirmed genetic risk factor of late onset AD, increases nuclear HDAC4 levels compared with the ApoE3 in transgenic mice (Sen et al., 2015). It suggests that increased HDAC4 expression or its nuclear localization may contribute to learning and memory deficits in patients with AD.

### HDAC4 in Frontotemporal Lobar Degeneration

Frontotemporal lobar degeneration (FTLD) is a heterogeneous neurodegenerative process resulting in frontotemporal dementia. Progressive difficulties in planning, organizing and language are the major characteristics of FTLD. The atrophy of frontal and temporal lobe and inclusions containing abnormal accumulation of Tau, TAR DNA binding protein (TDP-43) or FUS RNA bindind protein (FUS) are the characteristic pathological features of FTLD.

In FTLD patients, cytoplasmic HDAC4 is increased in granule cells of the dentate gyrus, while HDAC5, the other member of class IIa HDACs, is not altered, suggesting that HDAC4 may have a specific role in the pathology of FTLD (Whitehouse et al., 2015).

### HDAC4 in Huntington’s disease

Huntington’s disease is a common autosomal dominant neurodegenerative disease, which is caused by the expansion of polyglutamine repeats in huntingtin (HTT) protein, named as mutant HTT (mHTT) (MacDonald et al., 1993; Myers et al., 1993; Kremer et al., 1994). The characteristic clinical features are chorea, progressive cognitive decline, and psychiatric symptoms, while the cognitive problem is often the earliest symptom in patients with HD (Walker, 2007). mHTT impairs fast axonal transport, disrupts mitochondrial function and inflammatory response and promotes apoptosis, which may contribute to the cognitive decline (Szebenyi et al., 2003; Beal and Ferrante, 2004; Trushina et al., 2004).

Growing evidence suggests that increased HDAC4 is implicated in HD pathology, such that reducing HDAC4 expression has beneficial effects. First, overexpression of miR-22 has a protective effect on mHTT model cells, which may be mediated by HDAC4 reduction as HDAC4 is a target gene of miR-22 (Jovicic et al., 2013). Second, HDAC4 interacts with microtubule associated protein 1S (MAP1S) resulting in MAP1S destabilization and reduction, subsequently suppressing the clearance of mHTT aggregates and potentiating the toxicity of mHTT to cultured cells (Yue et al., 2015). Moreover, HDAC4 is associated with HTT in a polyglutamine-length-dependent manner and co-localized with cytoplasmic aggregates. However, reducing HDAC4 expression delays the formation of cytoplasmic aggregates, restores BDNF expression, and rescues synaptic dysfunction in HD mouse models (Mielcarek et al., 2013). In addition, suberoylanilide hydroxamic acid (SAHA) promotes HDAC4 degradation, suggesting that reducing HDAC4 expression may contribute to SAHA’s rescue effects on HD model mice via multiple HDAC4-associated pathways (**Figure 1**) (Mielcarek et al., 2011). However, SAHA is also an inhibitor of class I HDACs and HDAC6, suggesting that its rescue effects may also be mediated by inhibiting the deacetylase activity of class I HDACs and HDAC6. Although Quinti et al. (2010) showed that the reduction of HDAC4 is associated with the progression of HD in HD model mice, Mielcarek et al. (2013) did not observe the reduction in same HD model mice. However, they found that reducing HDAC4 expression has beneficial effects on HD mice (Mielcarek et al., 2013). As the alteration of HDAC4 in HD model mice remains inconclusive and it still lacks the evidence from HD patients, the alteration of HDAC4 in HD and its role in the pathology of HD need to be further investigated.

### HDAC4 in Parkinson Disease

Parkinson disease is the second most common neurodegenerative disease in the elderly. In addition to tremor, rigidity, gait disturbances etc. motor dysfunctions, PD patients also have cognitive impairments (Jankovic, 2008). The major pathological hallmark of PD is the Lewy bodies which mainly consist of protein aggregates of a-synuclein, parkin, and ubiquitin (Jellinger, 2009). In addition, same pathological features were observed in patients with Lewy body (LB) dementia (Jellinger, 2009).

A couple of studies indicate that HDAC4 is associated with the pathology of PD. First, mutations in the *Parkin* gene cause early onset familial PD and the dysregualtion of parkin has also been observed in sporadic PD. Second, parkin controls the levels of sumoylated HDAC4 (Kirsh et al., 2002; Um et al., 2006). Moreover, HDAC4 co-localized with α-synuclein in the LB (Takahashi-Fujigasaki and Fujigasaki, 2006). In addition, paraquat, a widely used herbicide, implicated in the induction of the pathology of PD, reduces the expression of HDAC4 in culture cells (Song et al., 2011). Furthermore, previous studies showed that aberrant HDAC4 expression results in learning and memory deficits in both mice and Drosophila (Kim et al., 2012; Fitzsimons et al., 2013). Above evidence suggests that alteration of HDAC4 may contribute to cognitive decline in patients with PD. However, no direct evidence shows that HDAC4 is implicated in the pathology of PD.

### HDAC4 in Ataxia-Telangiectasia

Ataxia-telangiectasia (A-T), a rare neurodegenerative disease, is caused by mutations in the *ATM* gene. A-T patients showed many premature aging components, characterized by difficulty in movement and coordination, and early cognitive impairment including learning and memory deficits (Vinck et al., 2011; Shiloh and Lederman, 2016).

In ATM deficient mice, nuclear HDAC4 is increased, which is mediated by the reduction of ATM-dependent phosphorylation of protein phosphatase 2A (PP2A). Reduced phosphorylation of PP2A results in increased HDAC4 dephosphorylation by enhancing PP2A-HDAC4 interaction (Li et al., 2012). HDAC4 dephosphorylation promotes its nuclear import and subsequent dysregulation of genes involved in synaptic plasticity, neuronal survival and neurodevelopment, which may contribute to the cognitive deficits in ATM mice. Consistently, reduced ATM accompanying with the increase of nuclear HDAC4 has been observed in brains of AD patients (Shen et al., 2016).

## Aberrant HDAC4 Expression/Function in Mental Disorders

Many mental disorders, including autism spectrum disorders (ASDs), depression, and schizophrenia, are associated with neurodevelopment defects and cognitive impairment is a core feature of mental disorders (Ronan et al., 2013). Increased evidence indicates that aberrant HDAC4 expression or function plays an important role in cognitive deficits of mental disorders (**Table 1**).

### HDAC4 in ASD and BMDR Syndrome

Autism spectrum disorder is characterized by the impairment of social and communication ability, as well as cognitive defects. Several lines of evidence suggests that dysregulation of HDAC4 is implicated in ASD (Pinto et al., 2014; Fisch et al., 2016). First, HDAC4 mRNA was significantly increased in autistic brains (Nardone et al., 2014). Moreover, ASD, intellectual disability, developmental delay etc. are the characteristics of Brachydactyly-mental-retardation (BDMR) syndrome which is caused by 2q37 microdeletion. Importantly, the *HDAC4* gene is located in this small region (Doherty and Lacbawan, 1993–2016). A rare case of BMDR syndrome carries an inactive mutant of HDAC4, suggesting that HDAC4 deficiency may be the cause of BMDR syndrome (Doherty and Lacbawan, 1993–2016; Williams et al., 2010). Moreover, in patients with BMDR syndrome, HDAC4 modulates the severity of symptoms in a dosage dependent manner, which further confirms the role of HDAC4 in ASD and other BMDR features (Morris et al., 2012).

### HDAC4 in Depressive Disorders

Depressive disorders are the most common mood disorder leading to disability, which is characterized by the presence of sad, empty, or irritable mood and cognitive impairment (Rock et al., 2014). Recent studies highly suggest that HDAC4 is implicated in the pathology of depressive disorders. First, aberrant expression of HDAC4 mRNA has been detected in patients with depression (Otsuki et al., 2012). Consistently, antidepressant reduces the recruitment of HDAC4 to the glial cell-derived neurotrophic factor (GDNF) promoter, consequently increasing the expression of GDNF which is reduced in patients with depression (Otsuki et al., 2012; Lin and Tseng, 2015). In patients with bipolar disorder (BPD), HDAC4 mRNA is significantly increased in a depressive state, while its expression is marked decreased in a remissive state (Hobara et al., 2010). In addition, HDAC4 mRNA is significantly increased in brains of forced-swim stress-induced- and postnatal fluoxetine-induced depression model mice (Sailaja et al., 2012; Sarkar et al., 2014). Intriguingly, adult fluoxetine application does not induce depression-like behavior in mice which is associated with unchanged HDAC4 expression. Ectopic overexpression of HDAC4 in hippocampus is sufficient to induce depression-like behavior in adult mice, indicating that HDAC4 elevation is the key to induce depression-like behavior (Sarkar et al., 2014). Furthermore, depression is a common feature in AD, which may be associated with the increase of HDAC4 expression in AD patients.

### HDAC4 in Schizophrenia

Schizophrenia is a complex psychiatric disorder, characterized by impairments in behavior, thought, and emotion. Cognitive impairment is common in patients with schizophrenia, in particular, learning and memory deficits. A couple of evidence suggests that HDAC4 might be associated with the pathology of schizophrenia. First, one SNP (rs1063639) in the *HDAC4* gene associates with the development of schizophrenia in a Korea population (Kim et al., 2010). Moreover, in patients with schizophrenia, HDAC4 mRNA is negatively associated with the expression of GAD67, a candidate gene of schizophrenia (Sharma et al., 2008). However, the exact role of HDAC4 in the cognitive deficits of schizophrenia needs to be further investigated.

## HDAC4, A Specific Target for Cognitive Impairment

Growing evidence indicates that HDAC4 is a specific target for the treatment of cognitive impairment in multiple disorders, which is different from other HDACs. First, HDAC4 is highly enriched in brain compared with other HDACs. Second, HDAC4 has no or weak HDAC activity, suggesting that global HDAC inhibitors, targeting the catalytic sites of HDACs, may have no effect on HDAC4’s function (Sando et al., 2012). Consistently, HDAC4 has a different effect on cognitive function compared with other HDACs. For example, conditional deletion of HDAC4 leads to learning and memory deficits, while global HDACs inhibition or HDAC2 deficiency significantly improves learning and memory in mice (Vecsey et al., 2007; Guan et al., 2009; Kim et al., 2012). Moreover, the maintenance of HDAC4 homeostasis is crucial for the disease treatment as either increased or decreased HDAC4 expression is detrimental to the cognitive function. It suggests that HDAC4 is a potential target for the treatment of cognitive impairment. However, only one selective HDAC4 inhibitor, tasquinimod, is commercially available, and its effect on cognitive function has not been explored. Therefore, specific HDAC4 modulators should be developed and their roles in cognitive disorders need to be investigated.

## Conclusion

Although HDAC4 belongs to the family of HDAC, its deacetylase activity is weak or undetectable. Thus, it remains elusive whether HDAC4 per se could repress gene transcription by its HDAC activity (**Figure 1**). However, HDAC4 could regulate the transcription of genes involved in synaptic plasticity, neuronal survival, and neurodevelopment by interacting with multiple proteins, which is essential for the maintenance of normal cognitive function (**Figure 1**). Moreover, HDAC4 may function to regulate protein SUMOylation via interacting with Ubc9 contributing to the maintenance of cognitive function (**Figure 1**). Moreover, aberrant expression of HDAC4 may be implicated in the cognitive impairment of neurodegenerative diseases and mental disorders. Therefore, HDAC4 is a potential therapeutic target to rescue cognitive deficits in above disorders.

## Author Contributions

YW: Formulated the study, wrote the manuscript, and designed the figure. FH, XW: Formulated the study and wrote the manuscript. QK, XH, BB: Provided intellectual thoughts, revised the manuscript, and project leaders.

## Conflict of Interest Statement

The authors declare that the research was conducted in the absence of any commercial or financial relationships that could be construed as a potential conflict of interest.
